# About the Performance of Latin American Gynecology and Obstetrics Journals in the International Scenario

**DOI:** 10.1055/s-0042-1744173

**Published:** 2022-02-25

**Authors:** Marcos Felipe Silva de Sá

**Affiliations:** 1 Editor-in-Chief – Revista Brasileira de Ginecologia e Obstetrícia – RBGO

In this issue, RBGO publishes a Letter to Editor 1 in which the author refers to the fact that Latin American Gynecology and Obstetrics Journals occupy low positions in the international rank of journals in the specialty and have little impact on the international scientific scenario. In his diagnosis to justify the low number of Latin American journals ranked in Scimago Journal Rankings (SJR) and the low citation rates of their articles, the author states that better quality articles are sent to international journals and there is little research training during medical residency, hence the authors have scientific limitations for this.

In fact, Latin American authors prefer to send their articles to international journals because of their greater visibility and projection. In the Brazilian case, additional factors of very relevant weight contribute to the international dispersion of our publications to the detriment of national journals. Regarding the lack of training in research during medical residency, in most countries, medical residency constitutes a differential for the training of professors in medicine. However, to become a professor/researcher of medicine in Brazil, especially in public universities, after performing the medical residency, the professional must attend a Postgraduate Program since medical residency programs are focused on preparing the physician for professional practice, and developing research and writing scientific texts is not part of their scope.

Postgraduate programs have two levels: Master or Doctoral studies. These programs are offered by few universities given the degree of requirements for their accreditation, done by a regulatory government agency, the Coordination for the Improvement of Higher Education Personnel - CAPES. Only a small portion of physician graduates from medical residency choose training in teaching and research in the Postgraduate Program. They study under guidance of a qualified professor (advisor), and for conclusion, students must prepare and defend a Master's or Doctoral thesis. For illustrative purposes, in 2021, 4,862 medical residency programs in different specialties were offered by 809 hospital institutions in Brazil in which 53,776 residents were enrolled. 2 3 On the other hand, considering the surgical and clinical areas, Master and Doctoral Postgraduate Programs are offered by 273 university institutions, where around 4,581 students were enrolled in 2021 (1,904 master and 2,677 doctoral students), corresponding only to 8.5% of the number of physicians enrolled in medical residency programs in Brazil. 4

Master and Doctoral Postgraduate Programs became a great source of medical research in Brazil, and their scientific production corresponds to an important aspect in the assessment of their quality performed periodically by CAPES. The quality of research is measured based on evaluation of journals where papers are published and the Impact Factor (IF) or the CiteScore of the journal are used as main criteria, establishing a ranking called the Qualis System. 5 In the development of Qualis, CAPES places Brazilian journals in direct competition with the main international journals, especially from the North America and Western Europe, which makes this dispute very unequal, with a clear disadvantage for Brazilian journals which end up stratified in the lowest percentiles of the classification. Evidently, this fact makes the publication in Brazilian journals uninteresting for the researchers, as these publications contribute nothing or little to the score in the evaluation of the Postgraduate Program. For these reasons, they are motivated to try to publish their theses in foreign journals with higher IF. In general, theses evidently are publications with better scientific quality and will have a greater chance of citations in the literature.

However, it has not been easy to publish in important international journals. They have very high demands for manuscript submissions, which implies high rejection rates, often even for well-qualified articles, as in many occasions, research can focus on an issue relevant to a particular community or country, but that may be irrelevant or uninteresting to readers of a foreign journal. In addition, it is important to mention the high fees in US dollars or euros charged by renowned international journals, resources often inaccessible to authors, which can lead them to give up publishing even the already accepted articles. After all, by Brazilian standards, the cost of a publication in some international journals may be the equivalent of developing the entire research project. In short, the end result of this whole context is the high percentage of good quality theses that end up not being published, which is a regrettable fact considering the investments made in research projects that will have their results abandoned in drawers of laboratories.

Despite all these difficulties, data presented in the charts of the Letter to Editor 1 show a Brazil with strong scientific production quantitatively and qualitatively, ahead of other countries in Latin America. Brazil currently occupies the 13 ^th^ position in the world in scientific publications and our universities stand out in the international scenario. Brazil is recognized as one of the countries that produce the most PhDs in the world and certainly already has the stature to be included in the list together to the main developed countries in research. Therefore, it would be natural for Brazil to have its own journals with international competitiveness. Unfortunately, Brazilian journals have not been the receptacles of much of the qualified science produced here due to the veiled incentive of CAPES for publications in foreign journals. CAPES seems not to believe in the potential of Brazilian journals to become internationally competitive in the short or medium term. Nowadays, ∼25–30% of articles published in RBGO, like in many others Brazilian journals, originate from authors from other countries, particularly in Latin America and Asia. So we must believe that we already have journals with international acceptance and potential to be competitive.

It is noteworthy that journals in the medical fields are maintained and financed by specialties societies themselves which are private and non-profit entities. The journals are edited as a result of the selfless work of their associates, who, in general, are professionals linked to universities and research institutions. They are the ones exercising functions of editors, reviewers, editorialists, etc., and almost all are teachers/advisors of Postgraduate Programs. All of them work voluntarily without any remuneration, trying to offer to Brazilian authors the opportunity to publish in journals of increasingly better quality and already with great international visibility. Therefore, it is difficult to understand how CAPES, which has on the advisors the support of the Postgraduate Programs, does not recognize these activities as an important part of the expression of national scientific production.

As a governmental Agency CAPES needs to make available competitive journals to internationally disseminate the significative Brazilian scientific production, especially those arising from the PPGs. It is necessary to create an opportunity to boost national journals and this has been a long-time request of Brazilian journals editors. The time has come for the Qualis System to be revised to become an instrument of a State policy to encourage Brazilian scientific literature. CAPES could establish the minimum quality criteria required for Brazilian journals based on international publishing standards and requiring them to be registered in the main international databases. Once they having fulfilled these requirements, each journal would have a established score designated on a scale attributed to Brazilian journals, without prejudice to the greater appreciation, in parallel, to international journals.

This way, the decision to publish papers in Brazil or abroad would be at researchers' free will, as they would always be contributing to increase the score of their respective Postgraduate Program. Everyone will have a gain: researchers (advisors and students), Postgraduate Programs, government funding agencies and Brazilian science as a whole, All this at zero cost to CAPES.

Therefore, dear readers, with regard to Brazilian journals, these are our explanations to answer the question: ‘Latin America Obstetrics and Gynecology. What is up with the journals?’. 1 Although the question was addressed to Latin American Gynecology and Obstetrics Journals, in the case of Brazil, the explanations are valid for journals of all specialties.
